# Differential adoption of castration‐resistant prostate cancer treatment across facilities in a national healthcare system

**DOI:** 10.1002/cam4.5490

**Published:** 2023-02-15

**Authors:** Megan E. V. Caram, Kyle Kumbier, Jennifer Burns, Jordan B. Sparks, Phoebe A. Tsao, Kristian D. Stensland, Samuel L. Washington, Brent K. Hollenbeck, Vahakn Shahinian, Ted A. Skolarus

**Affiliations:** ^1^ Department of Internal Medicine University of Michigan Medical School Michigan Ann Arbor USA; ^2^ VA Health Services Research & Development, Center for Clinical Management and Research, VA Ann Arbor Healthcare System Michigan Ann Arbor USA; ^3^ Department of Urology University of Michigan Medical School Michigan Ann Arbor USA; ^4^ Department of Urology University of California San Francisco California San Francisco USA; ^5^ Department of Urology University of Chicago Pritzker School of Medicine Illinois Chicago USA

**Keywords:** adoption of technology, facility, prostate cancer, rural care, variation in care

## Abstract

**Background:**

Over the past decade, abiraterone and enzalutamide have largely replaced ketoconazole as oral treatments for castration‐resistant prostate cancer (CRPC). We investigated the differential adoption of abiraterone and enzalutamide across facilities in a national healthcare system to understand the impact a facility has on the receipt of these novel therapies.

**Methods:**

Using data from the VA Corporate Data Warehouse, we identified a cohort of men with CRPC who received the most common first‐line therapies: abiraterone, enzalutamide, docetaxel, or ketoconazole between 2010 and 2017. We described variability in the adoption of abiraterone and enzalutamide across facilities by time period (2010–2013 or 2014–2017). We categorized facilities depending on the timing of adoption of abiraterone and enzalutamide relative to other facilities and described facility characteristics associated with early and late adoption.

**Results:**

We identified 4998 men treated with ketoconazole, docetaxel, abiraterone, or enzalutamide as first‐line CRPC therapy between 2010 and 2017 at 125 national facilities. When limiting the cohort to oral therapies, most patients treated earlier in the study period (2010–2013) received ketoconazole. A dramatic shift was seen by the second half of the study period (2014–2017) with most men treated with first‐line abiraterone (61%). Despite this shift and a new standard of care, some facilities persisted in the widespread use of ketoconazole in the later period, so‐called late adopting facilities. After multivariable adjustment, patients who received treatment at a late adopting facility were more likely receiving care at a lower complexity, rural facility, with less urology and hematology/oncology workforce (all *p* < 0.01).

**Conclusion:**

Many facilities persisted in their use of ketoconazole as first‐line CRPC therapy, even when other facilities had adopted the new standard of care abiraterone and enzalutamide. Further work is needed to identify the effect of this late adoption on outcomes important to patients.

## Graphical abstract

There is variability in the timing of adoption of novel oral therapies for castration resistant prostate cancer across different facilities.

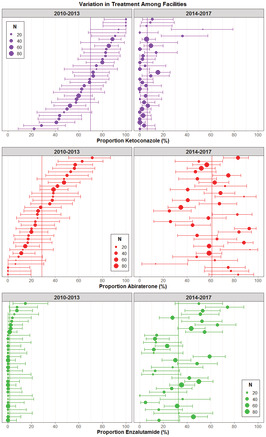

## 1 INTRODUCTION

Over 30,000 men die of prostate cancer annually in the US, most from end‐stage castration‐resistant prostate cancer (CRPC).^1^ Prior to 2011, oral ketoconazole was the most common first‐line CRPC treatment, used twice as often as docetaxel,^2^, ^3^, ^4^ though with no high‐level evidence supporting survival benefits. Since 2011, abiraterone and enzalutamide have become the most commonly used first‐line treatments for men with metastatic CRPC, largely replacing ketoconazole in first‐line settings as well as relegating docetaxel use toward less hormone‐responsive disease.^4^, ^5^, ^6^, ^7^, ^8^ This rapidly changing CRPC treatment landscape raises important questions regarding determinants of new technology adoption for men with advanced prostate cancer, especially determinants related to the facility and clinician prescribing the therapy.

Similar to differential adoption of new technology for other medical interventions,^9^, ^10^, ^11^, ^12^ patient and facility characteristics could have affected the adoption of abiraterone and enzalutamide, highlighting opportunities to learn about determinants and implications of sluggish adoption. For example, the extent to which facility characteristics (e.g., oncology staffing) impact the adoption of new CRPC treatments is currently unknown, though actionable for future treatments. Alternatively, whether facilities serving a greater proportion of Black vs. White men with CRPC have differential adoption provides insights into possible structural barriers to care. External factors that affect a patient's access to new therapies will become even more important in the coming years as the standard of care continues to shift and the use of abiraterone and enzalutamide moves earlier in the disease continuum.

In this context, we investigated the temporal adoption of newer oral CPRC treatments, abiraterone and enzalutamide, and the persistence of ketoconazole across facilities in a national healthcare system. We characterized facility determinants of early and late adoption of new oral treatments, and persistent use of ketoconazole. Understanding how external factors, such as the environment (facility) in which a patient is treated, can impact a patient's treatment is critical to efforts toward improving equitable access to novel therapies. To improve access to therapy, we must first understand potentially targetable systemic factors that would impact differential and delayed adoption of newer CRPC treatments in a national healthcare system.

## 2 METHODS

### 2.1 Cohort identification and treatment groups

We used data from the Veterans Health Affairs (VA) national healthcare system to conduct this study. The VA spans 130 local healthcare systems within 18 regional networks located across the entire US. Using data from the VA Corporate Data Warehouse harboring aggregated claims, pharmacy, laboratory, and medical record data from all VA facilities, we constructed a cohort of men receiving first‐line CRPC treatment, ketoconazole, docetaxel, abiraterone, or enzalutamide within the VA system from 2010 to 2017. We only included men with a diagnosis code for prostate cancer using the International Classification of Diseases, Ninth Revision (ICD‐9) code 185 for 2010–2015, and ICD‐10 code C61 for 2016 and 2017.

Next, we confirmed men had CRPC using two criteria. For 6 months prior to their first CRPC therapy, men were required to have (1) receipt of continuous androgen deprivation therapy (ADT) as defined previously^4^ and (2) a rising prostate‐specific antigen (PSA) level while on ADT. We defined a rising PSA on ADT as a PSA level prior to their initial CRPC drug that was greater than the most recent level, indicating castration resistance. Because testosterone was rarely measured in the laboratory data, we were unable to confirm castration. However, the use of a CRPC drug is consistent with clinical concern for castration resistance during this period of study. To ensure that the treatment received was the first‐line treatment for CRPC, we excluded patients who had received other CRPC therapies during the 6 months before starting CRPC therapy. For example, patients whom we identified as receiving ketoconazole but also received docetaxel within the 6 months prior to starting ketoconazole would have been excluded.

Patients were then grouped according to which first‐line CRPC therapy they received: docetaxel, ketoconazole, abiraterone, or enzalutamide. Although docetaxel was included in our cohort identification, we limited our primary analysis to include only those patients started on first‐line oral therapies abiraterone, enzalutamide, or ketoconazole to limit confounding variables such as patient and disease characteristics that impacted docetaxel use first line. In addition, facility logistics such as the availability of an infusion center and oncology nursing staff (variables not available to us) would impact the use of docetaxel first‐line but would not impact the use of oral therapies. We included all patients, including those who received docetaxel first‐line, in sensitivity analyses.

### 2.2 Patient and facility factors

We collected patient‐level data including age at first‐line oral CRPC therapy, comorbidity in the year prior, and race. We included disease‐status variables, such as PSA levels and doubling time since these have a small effect on the choice of first‐line oral therapy selection.^4^ We included a validated metastatic variable that became available after our cohort was constructed so that we could compare the presence of metastatic disease among patients started on the different first‐line therapies.^13^ We also included a rurality measure and distance to the treating facility as a measure of access.^14^, ^15^, ^16^

We then assigned patients to the VA facility providing their CRPC treatment. We assessed facility characteristics likely to impact the adoption of newer treatments (i.e., abiraterone and enzalutamide), and conversely, the persistence of an older oral therapy without evidence for benefit (i.e., ketoconazole). First, we used a standardized measure of VA facility complexity ranging from 1 (complex/tertiary) to 3 (primarily outpatient). As specialty care workforce staffing would be expected to align with the early adoption of advanced treatments, we also included the number of clinical full‐time equivalents (FTEs) of hematology/oncology and urology providers. For these providers, we included standardized annual patient volumes. Finally, we included a facility variable indicating the proportion of Black patients with CRPC to examine potential structural barriers to early adoption of advanced prostate cancer treatments.^17^, ^18^, ^19^, ^20^

We considered adopting facilities as those with at least one patient who received a prescription for abiraterone and/or enzalutamide between 2010 and 2017. Among adopters, we divided facilities into four phenotypes based on timing: (1) *early adopters* included those facilities that were ahead of the median in adopting both abiraterone and enzalutamide, (2) *late adopters* included facilities that were behind the median for adopting both, (3) *abiraterone preference* included facilities before the adoption median of abiraterone but behind the adoption median for enzalutamide, and last, (4) *enzalutamide preference* as the converse. Finally, we divided the study into two periods (2010–2013 and 2014–2017) to better characterize the adoption of newer agents (abiraterone, enzalutamide) and the persistence of older agent, ketoconazole. Abiraterone was approved for first‐line use in 2012 and enzalutamide for first‐line use in early 2014, so we expected ketoconazole to be most commonly used first line in the earlier timeframe and abiraterone and enzalutamide to be most commonly used in the later time period. We used caterpillar plots to show the proportion of patients receiving ketoconazole, abiraterone, or enzalutamide as first‐line treatment during these two periods.

### 2.3 Statistical analyses

For our main analysis, our primary outcome was patient receipt of an oral CRPC therapy at an early adopting facility. Given our primary outcome variable was not rare, we used Poisson regression models with robust standard errors to report rate ratios (RR) with confidence intervals (CI) to describe the association of patient and facility characteristics with being treated at an early adopting facility. A sensitivity analysis was conducted that included those patients who received first‐line docetaxel, which we expected to increase the sample size at several of the facilities but hypothesized would be unlikely to change the final results.

We used R version 4.1.2 and STATA version 16.0 for our analyses. This study followed the Strengthening the Reporting of Observational Studies in Epidemiology guideline for cohort studies.^21^ This study was approved by the Veterans Affairs Ann Arbor Healthcare System Internal Review Board.

## 3 RESULTS

**FIGURE 1:**
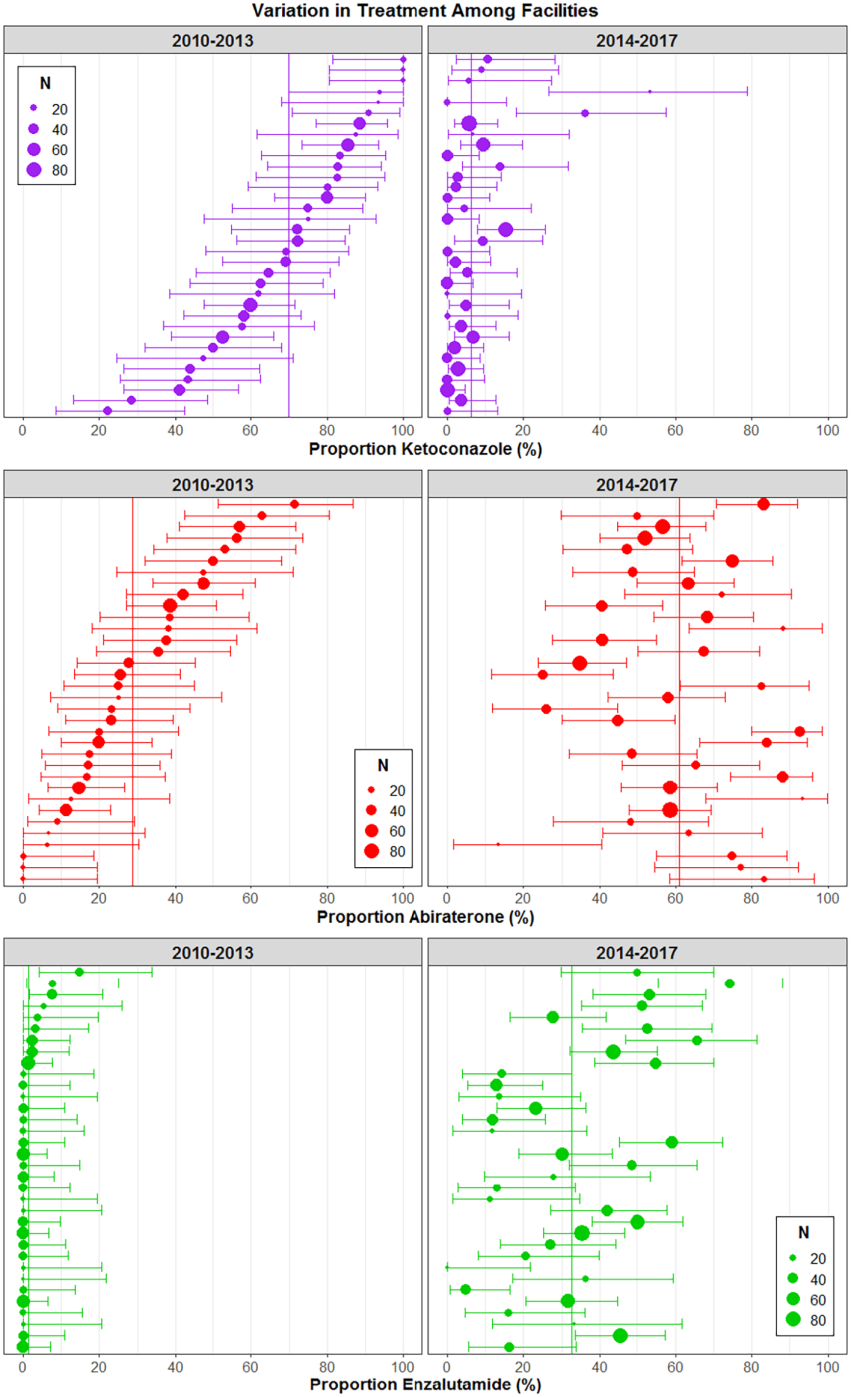
Caterpillar plots showing the distribution of facilities according to the proportion of patients receiving ketoconazole, abiraterone, or enzalutamide as first‐line therapy for castration‐resistant prostate cancer during two time periods. The vertical line in each panel represents the overall mean proportion of patients for that therapy across facilities, and the error bars represent the 95% confidence intervals within facilities. The size of the circle representing the mean proportion for each facility illustrates the number of patients treated at that facility. Only facilities with at least 15 patients treated in each time period were included, totaling 34 facilities. The horizontal position of a facility from 2010–2013 to 2014–2017 is the same within a medication panel, but the rank order of the facilities that was done according to their use of the therapy in the earlier time frame changes by the drug. Thus, the top row in the ketoconazole panels represents a different facility than the top row in the other medication panels.

Our original cohort included 4998 men with CRPC treated with CRPC therapy between 2010 and 2017 across 125 national facilities; 4122 of those men were treated with oral therapy. We identified minimal, clinically insignificant differences in patient and facility characteristics across different first‐line oral CPRC treatments over the entire study period (Table 1). Among the 34 facilities treating at least 15 CRPC patients with oral therapies during both time periods, 70% were treated with ketoconazole during the first study period (2010–2013), 29% with abiraterone, and 1% with enzalutamide. This pattern shifted dramatically during the second study period (2014–2017) so that the most commonly used oral therapy was abiraterone (61%), with one‐third of patients receiving enzalutamide (33%), and 6% ketoconazole. Some facilities persisted in their use of ketoconazole, even in the later period, with two facilities prescribing first‐line ketoconazole for over one‐third of CRPC patients (Figure 1).

**TABLE 1:** Patient and facility characteristics among men treated with oral castration‐resistant prostate cancer between 2010 and 2017 (*N* = 4122)

Characteristic	Abiraterone (*n* = 2073)	Enzalutamide (*n* = 936)	Ketoconazole (*n* = 1113)	*p*‐value (A vs E)	*p*‐value (A&E vs K)
Patient characteristics					
Age (median, IQR)	74 (67, 82)	75 (69, 83)	75 (67, 82)	**<0.01**	0.80
Race				0.06	0.50
White	1377 (67%)	586 (63%)	693 (62%)		
Black	565 (27%)	290 (31%)	313 (28%)		
Other	30 (1%)	18 (2%)	23 (2%)		
Unknown	101 (5%)	42 (4%)	84 (8%)		
Comorbidities				**<0.01**	**0.03**
0	1156 (56%)	462 (49%)	649 (58%)		
1	468 (22%)	204 (22%)	223 (20%)		
2+	449 (22%)	270 (29%)	241 (22%)		
Starting PSA ng/ml (median, IQR)	40 (15, 119)	30 (12, 88)	37 (14, 103)	**<0.01**	0.90
PSA doubling time				0.09	**0.02**
<3 months	111 (5%)	44 (5%)	42 (4%)		
3–6 months	870 (42%)	386 (41%)	428 (38%)		
6–10 months	736 (36%)	371 (40%)	462 (42%)		
>10 months	356 (17%)	135 (14%)	181 (16%)		
Metastatic at the start of treatment by NLP	1731 (84%)	784 (84%)	858 (77%)	0.90	**<0.01**
Start year (row %)				**<0.01**	**<0.01**
2010	0 (0%)	0 (0%)	266 (100%)		
2011	58 (13%)	0 (0%)	376 (87%)		
2012	154 (38%)	1 (0%)	248 (62%)		
2013	356 (73%)	21 (4%)	115 (23%)		
2014	420 (75%)	87 (15%)	58 (10%)		
2015	351 (58%)	229 (37%)	28 (5%)		
2016	348 (55%)	277 (43%)	12 (2%)		
2017	386 (54%)	321 (45%)	10 (1%)		
Distance to a facility in miles (median, IQR)^a^	24 (9, 58)	22 (9, 58)	25 (10, 64)	0.50	0.10
Rural or Urban^b^				0.40	0.40
Rural	697 (34%)	332 (35%)	397 (36%)		
Urban	1373 (66%)	604 (65%)	715 (64%)		
Facility characteristics					
Proportion Black (row %)				**<0.01**	0.09
Q4 (19.4%–49%) (*n* = 1403)	640 (45%)	362 (26%)	401 (29%)		
Q3 (9.0%–19.4%) *n* = 1139)	618 (54%)	245 (22%)	276 (24%)		
Q2 (3.3%–9.0%) (*n* = 961)	507 (52%)	189 (20%)	267 (28%)		
Q1 (0.2%–3.3%) (*n* = 617)	308 (50%)	140 (23%)	169 (27%)		
Facility complexity^c^				0.50	0.06
1	1761 (86%)	770 (83%)	954 (86%)		
2	211 (10%)	115 (12%)	124 (11%)		
3	90 (4%)	46 (5%)	32 (3%)		
Hem/Onc FTE (median, IQR)	2.63 (1.77, 3.68)	2.48 (1.77, 3.55)	2.63 (1.77, 3.84)	**0.04**	0.60
Urology FTE (median, IQR)	2.17 (1.66, 3.57)	2.03 (1.44, 3.08)	2.23 (1.70, 3.28)	**<0.01**	0.30
Hem/Onc‐patient ratio (median, IQR)	16 (14, 18)	16 (14, 18)	16 (14, 18)	0.60	0.80
Urology‐patient ratio (median, IQR)	9 (7, 11)	8 (7, 10)	8 (7, 11)	0.90	**0.02**

*Note*: HemOnc‐/Urology‐Patient Ratio: The number of Hematology/Oncology or Urology full‐time equivalents per 10,000 Hematology/Oncology or Urology patients.

Significant results are bolded.

Abbreviations: IQR, interquartile range; PSA, prostate‐specific antigen; A, abiraterone; E, enzalutamide; K, ketoconazole; Hem/Onc, hematology/oncology; FTE, full‐time equivalents.

^a^ There were a total of 61 patients with unknown distance traveled to the treating facility.

^b^ There were three abiraterone patients and one ketoconazole patient with missing Rural/Urban information.

^c^ Facility complexity ranges from 1 (complex/tertiary) to 3 (primarily outpatient). Eleven sites with a total of 19 patients were missing workforce data, so these patients did not contribute to the HemFTE/Ratio, UroFTE/Ratio, complexity.

**FIGURE 2:**
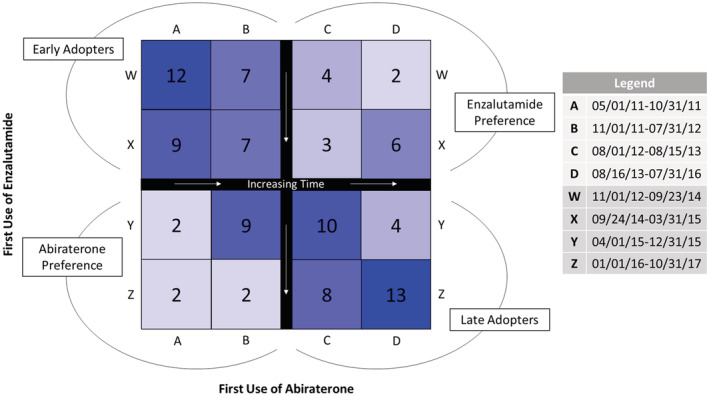
Characterization of facilities based on the timing of adoption of newer agents, abiraterone, and enzalutamide. The facilities were divided into quartiles by timing of adoption; for example, those facilities to the left or above the black divider line adopted the therapy earlier than the median. Most facilities were either Early Adopters or Late Adopters of both. The letters labeling the quartiles of the figure indicate the dates on which the first patient at that facility was prescribed abiraterone or enzalutamide. For example, patients treated at facilities in the A column first started receiving abiraterone between 5/1/2011 and 10/31/2011, while patients treated at facilities in the D column first started receiving abiraterone between 8/16/2013 and 7/31/2016. Only facilities that utilized both abiraterone and enzalutamide at some point were included.

We found 100 facilities that prescribed both abiraterone and enzalutamide at some point in the study timeframe. As illustrated in Figure 2, we characterized 35 facilities as early adopters, 35 facilities as late adopters, 15 as abiraterone preference, and 15 as enzalutamide preference facilities. We only compared late adopting facilities with early adopting facilities. Compared with early adopting facilities, late adopting facilities had a higher proportion of White patients (80% White vs. 63% White at early adopting, *p* < 0.01) and a higher proportion of patients who lived in rural settings (40% rural vs. 32% rural at early adopting, *p* < 0.01) (Table 2). There were no appreciable differences in disease characteristics among patients treated at late vs. early adopting facilities. The late adopting facilities tended to be lower complexity (*p* < 0.01) with less hematology/oncology workforce compared with early adopting facilities (2.2 FTE, 95% CI 1.7–3.0 vs. 2.8 FTE, 95% CI 2.2–4.8, *p* < 0.01). Finally, when characterizing facilities by their overall patient population, late adopting facilities had the lowest proportion of Black patients. For example, 14% of late adopting facilities were in the highest quartile of Black composition vs. 42% of early adopting facilities (*p* < 0.01).

**TABLE 2:** Patient and facility characteristics according to early and late adoption of oral castration‐resistant prostate cancer therapy in a national healthcare system

Characteristic	Facility phenotype^a^		
	Early adopter	Late adopter	*p*‐value^b^ (early vs late)
Patient characteristics	(*n* = 2225)	(*n* = 799)	
Age (median, IQR)	74 (67, 82)	75 (68, 83)	0.10
Race (%)			**<0.01**
White	1333 (60%)	600 (75%)	
Black	734 (33%)	135 (17%)	
Other	37 (2%)	16 (2%)	
Unknown	121 (5%)	48 (6%)	
Comorbidities			0.80
0	1229 (55%)	451 (56%)	
1	470 (21%)	165 (21%)	
2+	526 (24%)	183 (23%)	
Starting PSA ng/ml (median, IQR)	39 (15, 114)	36 (13, 98)	0.12
PSA doubling time			>0.90
<3 months	96 (4%)	36 (5%)	
3–6 months	935 (42%)	331 (41%)	
6–10 months	838 (38%)	309 (39%)	
>10 months	356 (16%)	123 (15%)	
Metastatic at the start of treatment by NLP	1811 (81%)	671 (84%)	0.10
Start year			**<0.01**
2010	129 (6%)	66 (8%)	
2011	250 (11%)	62 (8%)	
2012	226 (10%)	60 (8%)	
2013	282 (13%)	72 (9%)	
2014	315 (14%)	102 (13%)	
2015	333 (15%)	124 (15%)	
2016	332 (15%)	149 (19%)	
2017	358 (16%)	164 (20%)	
Distance to the facility in miles (median, IQR)^c^	24 (9, 60)	24 (8, 65)	0.70
Rural or Urban^d^			**<0.01**
Rural	718 (32%)	318 (40%)	
Urban	1504 (68%)	481(60%)	
Facility characteristics	(*n* = 35)	(*n* = 35)	
Proportion black			**<0.01**
Q4 (19.4%–49%)	933 (42%)	109 (14%)	
Q3 (9.0%–19.4%)	614 (28%)	259 (32%)	
Q2 (3.3%–9.0%)	478 (21%)	155 (19%)	
Q1 (0.2%–3.3%)	200 (9%)	276 (35%)	
Facility complexity^e^			**<0.01**
1	2117 (95%)	607 (76%)	
2	85 (4%)	111 (14%)	
3	23 (1%)	78 (10%)	
Hem/Onc FTE (median, IQR)	2.78 (2.17, 4.76)	2.19 (1.68, 3.03)	**<0.01**
Urology FTE (median, IQR)	2.15 (1.70, 3.81)	2.35 (1.41, 3.05)	**<0.01**
Hem/Onc‐patient ratio (median, IQR)	16 (14, 19)	16 (11, 19)	**<0.01**
Urology‐patient ratio (median, IQR)	8 (7, 10)	9 (7, 12)	**<0.01**

*Note*: HemOnc‐/Urology‐Patient Ratio: The number of Hematology/Oncology or Urology full‐time equivalents per 10,000 Hematology/Oncology or Urology patients.

Abbreviations: IQR, interquartile range; PSA, prostate‐specific antigen; Hem/Onc, hematology/oncology; FTE, full‐time equivalents.

^a^ Facilities not included in this table were abiraterone preference and enzalutamide preference. In addition, 170 patients were not included when categorizing facilities into a phenotype because they received care at sites that we were unable to classify into one of the four phenotypes.

^b^ Null Hypothesis: the characteristic is similar in both Early and Late Adopters; Alternative Hypothesis: the characteristic is different between the Early and Late Adopters. Significant results are bolded.

^c^ There were 60 patients who were excluded from the distance calculations as they had unknown distance traveled to the treating facility.

^d^ There were three patients at Early Adopting facilities who were excluded from the calculations as Rural/Urban characteristics were missing.

^e^ Facility complexity ranges from 1 (complex/tertiary) to 3 (primarily outpatient). One site with three patients is missing workforce data, so these patients did not contribute to the HemFTE/Ratio, UroFTE/Ratio, and complexity.

On univariable analysis, patients treated at lower complexity facilities were 2–3 times as likely to be at a late adopting facility (Table 3). Both medical oncology and urology workforce were associated with whether patients received treatment at a late adopting facility—greater FTEs were associated with early adopting facilities. Black patients were half as likely to receive treatment at a late adopting facility (rate ratio (RR) 0.50, 95% confidence interval 0.42–0.59) compared with White patients. However, this association was extinguished completely when adjusting for facility characteristics such as facility complexity and workforce suggesting that most of the association we see with the race on what treatment a patient will receive is due to the types of facilities where patients are treated.

**TABLE 3:** Patient and facility characteristics associated with late facility adoption of new oral CRPC treatments from 2010 to 2017 in a national healthcare system

		Rate ratio (RR) (95% CI) Late vs early adopters	
Independent variables		Unadjusted RR	Adjusted RR
Patient characteristics	Age (years)	1.01 (1.00, 1.01)	1.00 (1.00, 1.01)
	Race		
	White	–	–
	Black	**0.50 (0.42, 0.59)**	0.91 (0.75, 1.10)
	Other	0.97 (0.64, 1.47)	0.96 (0.70, 1.33)
	Comorbidities		
	0	–	–
	1	0.97 (0.83, 1.13)	0.99 (0.86, 1.14)
	2+	0.96 (0.83, 1.11)	1.00 (0.86, 1.15)
	Starting PSA (log scale)	0.97 (0.94, 1.01)	0.99 (0.95, 1.03)
	PSA doubling time		
	<3 months	–	–
	3–6 months	0.96 (0.71, 1.29)	0.90 (0.68, 1.18)
	6–10 months	0.99 (0.74, 1.33)	0.89 (0.68, 1.18)
	>10 months	0.94 (0.69, 1.29)	0.92 (0.68, 1.24)
	Metastatic at start	1.14 (0.97, 1.35)	1.14 (0.98, 1.32)
	Distance to facility (per 10 miles)	1.00 (1.00, 1.01)	1.00 (0.99, 1.01)
	Urban or Rural		
	Urban	–	–
	Rural	**1.27 (1.12, 1.43)**	0.89 (0.78, 1.01)
Facility characteristics	Proportion Black		
	Q4 (19.4%–49%)	–	–
	Q3 (9.0%–19.4%)	**2.84 (2.31, 3.48)**	**2.74 (2.15, 3.50)**
	Q2 (3.3%–9.0%)	**2.34 (1.87, 2.93)**	**2.13 (1.65, 2.76)**
	Q1 (0.2%–3.3%)	**5.54 (4.57, 6.73)**	**3.77 (2.86, 4.96)**
	Facility complexity^a^		
	1	–	–
	2	**2.54 (2.21, 2.93)**	1.16 (0.95, 1.43)
	3	**3.47 (3.05, 3.94)**	**1.45 (1.14, 1.86)**
	Hem/Onc FTE (per unit)	**0.71 (0.68, 0.74)**	**0.91 (0.86, 0.96)**
	Urology FTE (per unit)	**0.81 (0.77, 0.85)**	**0.90 (0.84, 0.97)**
	Hem/Onc‐Patient Ratio (per unit)	1.00 (0.99, 1.01)	1.00 (1.00, 1.00)
	Urology‐Patient Ratio (per unit)	**1.10 (1.07, 1.12)**	**1.07 (1.04, 1.10)**

*Note*: HemOnc‐/Urology‐Patient Ratio: The number of Hematology/Oncology or Urology full‐time equivalents per 10,000 Hematology/Oncology or Urology patients.

The unadjusted risk ratios were obtained from multiple univariate Poisson regression models. The Adjusted RRs were obtained from a single multivariate Poisson regression model. 95% CIs were calculated using robust standard errors. Significant results are bolded.

Abbreviations: RR, risk ratio; PSA, prostate‐specific antigen; A, abiraterone; E, enzalutamide; K, ketoconazole; Hem/Onc, hematology/oncology; FTE, full‐time equivalents.

^a^ Facility complexity ranges from 1 (complex/tertiary) to 3 (primarily outpatient).

A sensitivity analysis was done including the patients who received first‐line docetaxel to determine whether any of our findings changed with the larger cohort size. Most patients who received docetaxel first line received it in the earlier 2010–2013 time period, but ketoconazole was still the most commonly used therapy during that time, used first line at almost twice the rate as docetaxel. Patients who received docetaxel first line at any time point were disproportionately treated in higher complexity centers and centers with more Black patients (Table S1). We found no substantial differences in our rate ratios for patients being treated at an early adopting facility when including the patients who received first‐line docetaxel (Table S2).

## 4 DISCUSSION

In this study, we found that first‐line oral CRPC therapy using abiraterone, enzalutamide, and ketoconazole shifted dramatically within and across the national VA healthcare system between 2010 and 2017. Most VA facilities had adopted the newer oral therapies abiraterone and enzalutamide by the latter time period, but there were several facilities that still persisted in their use of ketoconazole first line. Higher complexity facilities and those with more cancer specialists were more likely to be early adopters of abiraterone and enzalutamide. Facilities that treated a higher proportion of Black patients were also more likely to be early adopting facilities. While this could be reflective of more advanced disease among Black patients, it may also represent institutional prioritization of early adoption to meet population needs. Taken together, national adoption of advanced prostate cancer treatment favored higher‐resourced, more complex facilities caring for urban, Black men.

We expected to identify treatment variation based on the emerging availability of new CRPC agents. For example, first‐line abiraterone was approved in 2012 and enzalutamide in 2014. However, we were not expecting to observe persistent use of first‐line ketoconazole in our later study period. These late adopting facilities tended to be lower in complexity and have a less specialist workforce, perhaps indicating fewer resources (e.g., pharmacies dispensing abiraterone/enzalutamide) or providers uncomfortable prescribing and managing newer medications. As a national integrated healthcare delivery system, communication and transfer of care between lower and higher complexity facilities are commonplace. Perhaps, follow‐up care for CRPC patients initiating treatment at late adopting, lower complexity facilities may need care coordination with higher complexity facilities to help ensure more timely adoption of evidence‐based care.

We found VA facilities serving a greater proportion of Black patients were more likely to be early adopting, independent of complexity level and whether patients lived in urban or rural settings. The effect of an individual patient's race on whether they were likely to receive care at a late adopting facility was mitigated completely when adjusting for the racial composition of the facility. This suggests the effect of race on treatment in the VA is mostly explained by the facility. The fact that early adopting facilities, hypothesized to be higher quality, tended to be facilities with more Black patients runs counter to what is commonly seen in community settings where community hospitals that disproportionately treat Black patients are usually lower quality with fewer resources and higher patient‐to‐physician ratios.^17^, ^20^ Our favorable findings of early access to advanced prostate cancer treatments for Black men should be made known more broadly for two reasons. First, in support of legislation further standardizing prostate cancer care across VA (i.e., H.R. 4880—117th Congress (2021–2022)). Second, a demonstration that national systems of care in the US can promote health equity even as our nation grapples with racial injustices.

As a national healthcare system, the VA achieves equivalent to superior health and delivery system outcomes across a variety of measures compared with the private sector. Additional resources provided by the VA to Veterans residing long distances from a medical center, including travel pay and hotel subsidies, may help mitigate challenges of distance and even urbanicity on quality of care and outcomes. The stakes for delivering high‐quality care have never been higher as the use of novel therapies abiraterone and enzalutamide move to earlier stages of metastatic disease when the cancer is still “hormone‐ or castration‐sensitive.” In the castration‐sensitive setting, abiraterone and enzalutamide have the potential to improve patient survival by years as opposed to months when given in the castration‐resistant setting.^22^, ^23^ Thus, understanding nonclinical facility‐level factors that influence treatments will be critical to ensuring equitable delivery of high‐quality care.

There are limitations in this analysis. First, the database we used includes a reliable variable that determines the presence of metastatic disease but does not characterize the extent of metastatic disease in CRPC patients. Therefore, it is possible the extent of metastatic disease may have been less severe in some facilities. We were able to determine that patients treated in facilities that were late adopters were similar in age, comorbidity, and disease characteristics (i.e., PSA level and doubling time) to those treated in early adopting sites, so it is possible that the other disease characteristics would account for most of the differences in the extent of metastatic disease. Second, the use of race as a proxy to identify potential structural barriers to care is limited since unmeasured factors that may explain the disparate outcomes among patients who are Black are not available in this database (e.g., environmental exposures, experience with racism). In addition, variables describing social determinants of health such as educational level and income, which are known to affect variation and are commonly associated with race and structural barriers to care were not available through the corporate data warehouse data set used for this study. Patient preference and quality of life are also important factors that can impact variation in care as well but were beyond the scope of this study.

In conclusion, we identified several facilities with persistent use of ketoconazole as first‐line therapy for men with CRPC, that is, late adopters, despite most others adopting abiraterone and enzalutamide. These trends mirror prior studies demonstrating variation in the diffusion of new technology where facilities in rural settings, with lower complexity and physician workforce, tend toward sluggish adoption. Early adopting facilities in the VA had higher proportions of Black patients potentially representing prioritization to meet population needs. The urgency of understanding variables that impact the adoption of novel therapies is critical as we move into an era where abiraterone and enzalutamide are recommended earlier in metastatic disease, for longer periods of time, and with greater potential for benefit.

## AUTHOR CONTRIBUTIONS

**Megan Elizabeth Veresh Caram:** Conceptualization (lead); funding acquisition (lead); investigation (lead); methodology (equal); resources (lead); supervision (lead); writing – original draft (lead); writing – review and editing (lead). **Kyle Kumbier:** Data curation (equal); formal analysis (equal); investigation (equal); methodology (lead); resources (equal); software (equal); validation (equal); writing – review and editing (equal). **Jennifer Burns:** Conceptualization (equal); data curation (lead); formal analysis (supporting); investigation (supporting); methodology (supporting); resources (equal); software (equal); writing – review and editing (equal). **Jordan B Sparks:** Investigation (supporting); project administration (lead); resources (supporting); supervision (equal); visualization (supporting); writing – review and editing (equal). **Phoebe A. Tsao:** Conceptualization (equal); formal analysis (equal); investigation (equal); methodology (equal); visualization (equal); writing – review and editing (equal). **Kristian D Stensland:** Conceptualization (equal); formal analysis (supporting); investigation (supporting); methodology (supporting); visualization (supporting); writing – review and editing (equal). **Samuel Lefridge Washington:** Conceptualization (supporting); investigation (supporting); visualization (supporting); writing – review and editing (supporting). **Brent K Hollenbeck:** Conceptualization (supporting); formal analysis (supporting); investigation (supporting); resources (supporting); supervision (equal); visualization (equal); writing – review and editing (equal). **Vahakn B Shahinian:** Conceptualization (supporting); formal analysis (supporting); investigation (supporting); resources (supporting); supervision (supporting); visualization (supporting); writing – review and editing (equal). **Ted Skolarus:** Conceptualization (equal); formal analysis (equal); funding acquisition (equal); investigation (equal); methodology (equal); project administration (equal); resources (equal); supervision (equal); visualization (equal); writing – review and editing (equal).

## CONFLICT OF INTEREST

The authors have no competing interests to disclose.

## Supporting information


Table S1.
Table S2.

## Data Availability

The data that support the findings of this study are not publicly available due to VA privacy restrictions on data.
