# Supplementary material for: Differential adoption of castration‐resistant prostate cancer treatment across facilities in a national healthcare system
Source: Cancer Med. 2023 Feb 15;12(6):6945–55. doi: 10.1002/cam4.5490 (PMC10067072; doi:10.1002/cam4.5490)
Supplement: Supplementary file 1 — Table S1. Table S2. [file CAM4-12-6945-s001.docx]

**Supplemental Table 1.** Patient and facility characteristics of patients started on first-line therapy (including abiraterone, enzalutamide, ketoconazole, or docetaxel) for castration resistant prostate cancer according to whether they were treated at an early adopting (rapid adopter of abiraterone and enzalutamide) or late adopting facility in a national healthcare system.

| **Characteristic** | **Facility phenotype^a^** | | |
| --- | --- | --- | --- |
|  | **Early adopter** | **Late adopter** | **p-value^b^**  **(Early vs Late)** |
| *Patient characteristics* | (n = 2680) | (n = 979) |  |
| Age (median, IQR) | 73 (66, 81) | 74 (67, 82) | 0.20 |
| Race (%) |  |  | **< 0.01** |
| White | 1597 (60%) | 729 (74%) |  |
| Black | 881 (33%) | 169 (17%) |  |
| Other | 44 (2%) | 20 (2%) |  |
| Unknown | 158 (6%) | 61 (6%) |  |
| Comorbidities |  |  | 0.90 |
| 0 | 1509 (56%) | 557 (57%) |  |
| 1 | 563 (21%) | 207 (21%) |  |
| 2+ | 608 (23%) | 215 (22%) |  |
| Starting PSA ng/mL (median, IQR) | 43 (15, 133) | 41 (14, 119) | 0.20 |
| PSA Doubling Time |  |  | >0.90 |
| <3 months | 135 (5%) | 50 (5%) |  |
| 3-6 months | 1139 (42%) | 416 (42%) |  |
| 6-10 months | 983 (37%) | 365 (37%) |  |
| >10 months | 423 (16%) | 148 (15%) |  |
| Metastatic at start of treatment by NLP | 2212 (83%) | 827 (84%) | 0.20 |
| Start Year |  |  | **< 0.01** |
| 2010 | 216 (8%) | 98 (10%) |  |
| 2011 | 336 (13%) | 102 (10%) |  |
| 2012 | 302 (11%) | 85 (9%) |  |
| 2013 | 313 (12%) | 96 (10%) |  |
| 2014 | 370 (14%) | 119 (12%) |  |
| 2015 | 366 (14%) | 140 (14%) |  |
| 2016 | 383 (14%) | 161 (16%) |  |
| 2017 | 394 (15%) | 178 (18%) |  |
| Distance to facility in miles  (median, IQR)^c^ | 24 (10, 61) | 26 (8, 65) | >0.90 |
| Rural or Urban^d^ |  |  | **< 0.01** |
| Rural | 865 (32%) | 384 (39%) |  |
| Urban | 1811 (68%) | 595 (61%) |  |
| *Facility characteristics* | (n=35) | (n=35) |  |
| Proportion Black |  |  | **< 0.01** |
| Q4 (19.4% - 49%) | 1123 (42%) | 129 (13%) |  |
| Q3 (9.0% - 19.4%) | 795 (30%) | 336 (34%) |  |
| Q2 (3.3% - 9.0%) | 554 (21%) | 205 (21%) |  |
| Q1 (0.2% - 3.3%) | 208 (8%) | 309 (32%) |  |
| Facility Complexity^e^ |  |  | **< 0.01** |
| 1 | 2560 (96%) | 759 (78%) |  |
| 2 | 93 (4%) | 125 (13%) |  |
| 3 | 27 (1%) | 92 (9%) |  |
| Hem/Onc FTE  (median, IQR) | 2.78  (2.17, 5.10) | 2.39  (1.68, 3.03) | **< 0.01** |
| Urology FTE  (median, IQR) | 2.14  (1.70, 3.81) | 2.35  (1.41, 3.05) | **< 0.01** |
| Hem/Onc-Patient ratio  (median, IQR) | 16  (14, 19) | 16  (11, 19) | **< 0.01** |
| Urology-Patient ratio  (median, IQR) | 8  (7, 10) | 9  (6, 12) | **< 0.01** |

Abbreviations: IQR, interquartile range; PSA, prostate specific antigen; ng/mL, nanograms/milliliter; Hem/Onc, hematology/oncology; FTE, full-time equivalents.

HemOnc-/Urology-Patient Ratio: The number of Hematology/Oncology or Urology full-time equivalents per 10,000 Hematology/Oncology or Urology patients.

^a^Patients not included in this table were those treated at facilities identified as abiraterone preference and enzalutamide preference (they were early adopting for one but late adopting for the other). In addition, 170 patients were not included when categorizing facilities into a phenotype because they received care at sites that we were unable to classify into one of the four phenotypes.

^b^Null Hypothesis: the characteristic is similar in both Early and Late Adopters; Alternative Hypothesis: the characteristic is different between the Early and Late Adopters. Significant results are **bolded**.

^c^There were 60 patients who were excluded from the distance calculations as they had unknown distance traveled to treating facility.

^d^There were three patients at Early Adopting facilities who were excluded from the calculations as Rural/Urban characteristics were missing.

^e^Facility complexity ranges from 1 (complex/tertiary) to 3 (primarily outpatient). One site with three patients is missing workforce data, so these patients did not contribute to the HemFTE/Ratio, UroFTE/Ratio, complexity.

**Supplemental Table 2.** Patient and facility characteristics associated with a patient being treated at a late adopting facility from 2010 through 2017 in a national healthcare system.

|  | | **Rate ratio (RR) (95% CI)**  **Late vs early adopters** | |
| --- | --- | --- | --- |
| **Independent Variables** | | **Unadjusted RR** | **Adjusted RR** |
| **Patient Characteristics** | Age (years) | 1.00 (1.00, 1.01) | 1.00 (1.00, 1.01) |
|  | Race |  |  |
|  | White | - | - |
|  | Black | **0.51 (0.44, 0.60)** | 0.92 (0.78, 1.08) |
|  | Other | 1.00 (0.69, 1.44) | 0.95 (0.70, 1.28) |
|  | Comorbidities |  |  |
|  | 0 | - | - |
|  | 1 | 1.00 (0.87, 1.14) | 1.02 (0.90, 1.15) |
|  | 2+ | 0.97 (0.85, 1.11) | 0.99 (0.87, 1.13) |
|  | Starting PSA (log scale) | 0.98 (0.95, 1.02) | 1.00 (0.97, 1.04) |
|  | PSA Doubling Time |  |  |
|  | <3 months | - | - |
|  | 3-6 months | 0.99 (0.77, 1.27) | 0.93 (0.73, 1.17) |
|  | 6-10 months | 1.00 (0.78, 1.29) | 0.93 (0.74, 1.18) |
|  | >10 months | 0.96 (0.73, 1.26) | 0.95 (0.73, 1.22) |
|  | Metastatic at Start | 1.11 (0.96, 1.29) | 1.13 (0.99, 1.30) |
|  | Distance to Facility  (per 10 miles) | 1.00 (1.00, 1.01) | 1.00 (0.99, 1.00) |
|  | Urban or Rural |  |  |
|  | Urban | - | - |
|  | Rural | **1.24 (1.12, 1.39)** | 0.90 (0.80, 1.01) |
| **Facility Characteristics** | Proportion Black |  |  |
|  | Q4 (19.4% - 49%) | - | - |
|  | Q3 (9.0% - 19.4%) | **2.88 (2.39, 3.47)** | **2.90 (2.32, 3.62)** |
|  | Q2 (3.3% - 9.0%) | **2.62 (2.14, 3.20)** | **2.43 (1.93, 3.07)** |
|  | Q1 (0.2% - 3.3%) | **5.80 (4.85, 6.93)** | **4.08 (3.16, 5.26)** |
|  | Facility Complexity^a^ |  |  |
|  | 1 | - | - |
|  | 2 | **2.51 (2.20, 2.86)** | 1.20 (1.00, 1.45) |
|  | 3 | **3.38 (3.01, 3.80)** | **1.50 (1.20, 1.87)** |
|  | Hem/Onc FTE (per unit) | **0.73 (0.71, 0.76)** | **0.94 (0.89, 0.98)** |
|  | Urology FTE (per unit) | **0.83 (0.80, 0.88)** | **0.91 (0.86, 0.97)** |
|  | Hem/Onc-Patient Ratio  (per unit) | 1.00 (1.00, 1.01) | 1.00 (1.00, 1.00) |
|  | Urology-Patient Ratio  (per unit) | **1.09 (1.06, 1.11)** | **1.06 (1.04, 1.09)** |
|  | | | |

Abbreviations: RR, risk ratio; PSA, prostate specific antigen; A, abiraterone; E, enzalutamide; K, ketoconazole; Hem/Onc, hematology/oncology; FTE, full-time equivalents.

HemOnc-/Urology-Patient Ratio: The number of Hematology/Oncology or Urology full-time equivalents per 10,000 Hematology/Oncology or Urology patients.

The Unadjusted risk ratios were obtained from multiple univariate Poisson regression models. The Adjusted RRs were obtained from a single multivariate Poisson regression model. 95% CIs were calculated using robust standard errors. Significant results are **bolded**.

^a^Facility complexity ranges from 1 (complex/tertiary) to 3 (primarily outpatient).
